# Hospital Obscurantism

**Published:** 1892-01-09

**Authors:** T. Fraser Black

**Affiliations:** 14, Mount-View Road, Crouch Hill, N.


### HOSPITAL OBSCURANTISM.

We have received from Mr. T. Fraser-Black, of Queen Victoria street and Crouch End, the following copy of a letter sent to the editor of the *Pall Mall Gazette* on the subject of its recent charges against voluntary hospitals. Our readers will probably not be surprised that, though it is dated December 29th, the editor of the *Pall Mall* has not yet permitted it to make its appearance in the columns of that newspaper :—

Sir,—Is it quite right that a newspaper professing such a high moral tone as the *P. M. G.* should empty its tar bucket when it only pretends to be giving a few dabs with the tar brush? In your article of the 26th, "What to do about Hospitals?" you condemn wholesale; you have not a good word to say for any hospital, and you do your very best to induce the public to keep their subscriptions in their pockets. Might I point out to you the fact that all hospitals even in London are not managed by old women, or even old fogies, who are perhaps a worse class than old women? May I tell you of one, the Great Northern Central Hospital (name to be given or withheld as you like), where the management is in the hands of business men, who are able to conduct their own businesses successfully, and who devote both their time and their energies, week by week, to see that the hospital is managed on the same business principles as are necessary to conduct the affairs of a successful newspaper or any other enterprise.

I don't suppose you can spare space for details, but if you like I can give you them, or if you will come and see for yourself you can then form an opinion, and if you think the place is properly managed you might present the institution with, say, £100, as a fine for your unjust and unfair wholesale remarks. T. Fraser Black.

14, Mount-View Road, Crouch Hill, N.
December 29th, 1891.

